# Periconceptional diet quality is associated with gestational diabetes risk and glucose concentrations among nulliparous gravidas

**DOI:** 10.3389/fendo.2022.940870

**Published:** 2022-09-05

**Authors:** Karen L. Lindsay, Gina F. Milone, William A. Grobman, David M. Haas, Brian M. Mercer, Hyagriv N. Simhan, George R. Saade, Robert M. Silver, Judith H. Chung

**Affiliations:** ^1^ Susan Samueli Integrative Health Institute, Susan & Henry Samueli College of Health Sciences, University of California, Irvine, CA, United States; ^2^ Division of Endocrinology, Department of Pediatrics, University of California, Irvine, School of Medicine, Orange, CA, United States; ^3^ Division of Maternal-Fetal Medicine, Department of Obstetrics & Gynecology, University of California, Irvine, School of Medicine, Orange, CA, United States; ^4^ Division of Maternal-Fetal Medicine, Department of Obstetrics and Gynecology, Feinberg School of Medicine, Northwestern University, Chicago, IL, United States; ^5^ Department of Obstetrics and Gynecology, Indiana University School of Medicine, Indianapolis, IN, United States; ^6^ Division of Maternal-Fetal Medicine, Department of Obstetrics and Gynecology, Cleveland, OH, United States; ^7^ Division of Maternal-Fetal Medicine, Department of Obstetrics, Gynecology, and Reproductive Sciences, University of Pittsburgh School of Medicine, Pittsburgh, PA, United States; ^8^ Division of Maternal-Fetal Medicine, Department of Obstetrics and Gynecology, University of Texas Medical Branch, Galveston, TX, United States; ^9^ Division of Maternal-Fetal Medicine, Department of Obstetrics and Gynecology, University of Utah Health Sciences Center, Salt Lake City, UT, United States

**Keywords:** periconception, pregnancy, alternative healthy eating index, diet quality, gestational diabetes mellitus, gestational glycemia, women’s health

## Abstract

**Background:**

Gestational diabetes mellitus (GDM) and elevated glucose concentrations below the threshold for GDM diagnosis have been associated with adverse pregnancy and offspring outcomes. Dietary interventions initiated during pregnancy have demonstrated inconsistent beneficial effects. Limited data exist regarding the effects of periconceptional diet on gestational glycemia.

**Objective:**

To evaluate independent associations between periconceptional diet quality with GDM frequency and glucose concentrations from GDM screening and diagnostic tests among nulliparous gravidas.

**Design:**

This is a secondary analysis of N=7997 participants from the NuMoM2b multicenter, prospective, observational cohort study of first pregnancies. The Alternative Healthy Eating Index (AHEI)-2010 was computed from food frequency questionnaires completed in early pregnancy (6-13 weeks), reporting usual dietary intake over the preceding 3 months. GDM screening was performed either by non-fasting 1-hour 50g glucose load (N=6845), followed by 3-hour 100g glucose tolerance test (GTT) for those with raised glucose concentrations (N=1116; at risk for GDM), or by a single 2-hour 75g GTT (N=569; all GDM risk levels). Logistic and linear regression were used to estimate the associations between the AHEI-2010 score with odds of GDM, having raised blood glucose on the 1-hour screening test, and continuous glucose concentrations on screening and diagnostic tests. All models were adjusted for *a priori* covariates: maternal age, race/ethnicity, early-pregnancy body mass index, smoking habits, rate of gestational weight gain, energy intake, nausea and vomiting in early pregnancy, study site.

**Results:**

Poorer periconceptional diet quality was observed among participants who were younger, with higher BMI, lower income levels, and of non-Hispanic Black or Hispanic ethnicity. The GDM rate was 4%. Each 1-point increase in AHEI-2010 score was associated with a 1% decrease in the odds of being diagnosed with GDM (beta=-0.015, p=0.022, OR=0.986, 95% CI 0.973 to 0.998). Diet quality was inversely associated with each post glucose load concentration on the non-fasting screening test and the 2-hour and 3-hour GTT.

**Conclusion:**

Poor periconceptional diet quality is independently associated with an increased risk of GDM and with minor elevations in serum glucose concentrations on GDM screening and diagnostic tests, in a diverse cohort of nulliparas. Periconception intervention studies targeting diet quality are warranted.

## Introduction

Glycemic control during pregnancy is an imperative component of prenatal care. Gestational diabetes mellitus (GDM) is a common complication of pregnancy, currently estimated to affect 63.5 per 1000 live births in the United States, and its prevalence is increasing across all racial/ethnic groups (1). GDM carries significant maternal and/or perinatal morbidity as affected pregnant individuals are more likely to develop preeclampsia or undergo a cesarean delivery, while later in life they have up to 70% odds of developing type 2 diabetes (2). Neonates are at increased risk for large for gestational age birth weight, birth trauma, hypoglycemia, as well as obesity and diabetes later in life (2). Further, modestly elevated glucose concentrations on glucose tolerance tests (GTT), even in the absence of overt GDM, have been associated with an increased risk of adverse pregnancy outcomes (3, 4). Interventions to decrease the incidence of GDM and milder hyperglycemic cases are warranted.

Excess gestational weight gain (GWG) is a known, modifiable risk factor for GDM (5). Although several randomized controlled trials have shown improved adherence to GWG guidelines with lifestyle interventions (6), this has not consistently been associated with improvement in perinatal outcomes (7–9). The lack of evidence surrounding prenatal lifestyle interventions has led investigators to explore other modifiable nutrition parameters, including prenatal diet quality. Although no diet has been shown to be the single best choice for all pregnancies (10), benefit has been seen with the Mediterranean diet, the low glycemic-index diet, and diets that emphasize plant-based rather than animal-based protein (11–14).

As GDM is typically diagnosed in the late second or early third trimester, it has been suggested that implementing dietary changes at this stage is too late to maximally impact pregnancy and neonatal outcomes (15). Greater attention is now being paid to the preconception period as a potentially efficacious window for health behavior change to support improved pregnancy outcomes (16).

Preconceptional diets higher in red meat and processed foods have been previously shown to be associated with an increased incidence of GDM (11–13). In a prior analysis from the Nulliparous Pregnancy Outcomes Study: Monitoring Mothers-To-Be (NuMoM2b) cohort that the present study utilizes, periconceptional diet quality, measured by the Healthy Eating Index (HEI), was associated with numerous adverse pregnancy outcomes, but not with GDM incidence (17). However, the effect of periconceptional diet on glycemia across a continuum, regardless of GDM diagnosis, has not yet been studied. Further, there is need for a more comprehensive characterization of periconceptional diet quality using an index that is strongly associated with chronic disease risk, such as the Alternative Healthy Eating Index (AHEI)-2010 (18, 19). The AHEI-2010 differs from the HEI in that it incorporates quantitative scoring for *qualitative* dietary guidelines (e.g., choose more fish, poultry, and whole grains, and if you drink alcohol, do so in moderation) that are specifically associated with reduced chronic disease risk, particularly diabetes and coronary heart disease (18).

The aim of this study was to determine the prospective association between periconceptional diet and frequency of GDM, as well as gestational glucose at the time of GDM screening and diagnostic testing, in a diverse cohort of nulliparas.

## Materials and methods

This is a secondary analysis of maternal dietary and glycemic data from the NuMoM2b cohort, a large, multicenter, prospective observational study conducted at 8 U.S. medical centers from 2010 to 2013. Each site’s local governing Institutional Review Board(s) approved the nuMoM2b protocol and procedures.

Individuals were eligible if they had a viable singleton pregnancy, had no prior pregnancy lasting ≥20 weeks’ gestation, and were between 6 + 0 to 13 + 6 weeks’ gestation at the time of enrollment. Exclusion criteria included age <13 years, history of ≥3 spontaneous abortions, likely fatal fetal malformation evident before enrollment, known fetal aneuploidy, assisted reproduction with a donor oocyte, multifetal reduction, or plan to terminate the pregnancy. Complete study protocol details have been previously published (20). Participants classified as having pre-gestational diabetes were excluded from the present analysis.

Diet was assessed by the validated modified Block 2005 Food Frequency Questionnaire (FFQ) at visit 1 (21, 22), when participants were between 6 + 0 to 13 + 6 weeks’ gestation. The Block FFQ assesses energy intake, 52 nutrients and 35 food groups from approximately 120 food and beverage items and includes serial adjustment items to estimate portion size. Participants were asked to report usual dietary intake over the preceding 3 months, thereby reflecting the periconceptional period.

The AHEI-2010, a validated predictor of chronic disease risk (19), was computed as a summary score of overall diet quality. The AHEI-2010 is comprised of 11 food group or nutrient components: vegetables, fruit, wholegrains, sugary beverages and fruit juice, red and processed meat, nuts and legumes, long-chain omega-3 fats, trans fatty acids, polyunsaturated fatty acids, sodium, and alcohol. Individuals are assigned a score from 0-10 for each component, where higher scores indicate greater compliance to recommended intakes of that food group or nutrient. Component scores are summed to give a total AHEI-2010 score ranging from 0-110. The specific criterion for scoring each component has been previously described (18).

Nausea and vomiting of pregnancy commonly occurs in the first trimester and may influence dietary intake periconceptionally. The validated Pregnancy-Unique Quantification of Emesis (PUQE) scale was completed by participants at the first study visit to assess the degree of nausea and vomiting experienced. This scale produces a continuous score from 3-15, with higher scores representing more severe nausea and vomiting.

Outcome variables for this analysis were serum glucose concentrations on 50g glucose screening test, fasting and post glucose load serum glucose concentrations from GTTs performed as part of routine clinical practice, and presence or absence of GDM abstracted from medical records based on local diagnostic criteria (2-hour 75g GTT or 3-hour 100g GTT). Specifically, for the 2-hour GTT, a diagnosis of GDM is established when any single threshold value is met or exceeded (fasting, 92 mg/dL; 1-hour, 180 mg/dL; or 2-hour, 153 mg/dL) (23). For the 3-hour GTT, GDM is diagnosed when two or more threshold values are met or exceeded (fasting, 95 mg/dL; 1-hour, 180 mg/dL; 2-hour, 155 mg/dL; 3-hour, 140 mg/dL) (24). Having an elevated glucose result on the 50g non-fasting glucose screening test, using the threshold of ≥140 mg/dL as recommended by the American College of Obstetricians and Gynecologists (2), was also considered as a secondary outcome measure as this is a widely used indicator of GDM risk. Glucose concentrations from GTTs were considered separately in analyses according to 2-hour or 3-hour testing method. For participants who had multiple GTTs performed during pregnancy, the glycemic concentrations from the test conducted closest to 26-28 weeks’ gestation were selected for this analysis, as this is the most widely accepted timepoint for routine GDM screening. Serum glucose concentrations were determined by enzymatic assay at each study site according to local protocols.

*A priori* covariates included in the analysis were selected based on their known association with GDM and impaired glycemia in pregnancy: maternal body mass index (BMI), age, self-reported race/ethnicity, self-reported smoking status within the prior 3 months (yes/no), rate of GWG until the approximate time of the GTT. Additionally we adjusted for energy intake and the PUQE score as these factors may influence the AHEI-2010 score. BMI was computed in early pregnancy using measured weight and height at enrollment, according to the formula weight (kg)/height (m)^2^. Rate of GWG per week was computed as the difference in maternal measured weight between the enrollment visit and study visit 3 (at 22 + 0 to 29 + 6 weeks’ gestation), divided by the number of weeks between measurement dates. Additionally, study site was entered to all models as a covariate to control for potential differences in glycemic concentrations on GDM screening and diagnostic tests due to different assay kits and laboratory techniques.

Statistical analyses were performed using IBM SPSS Statistics version 26. The AHEI-2010 score was described continuously and categorically by quartiles. Descriptive statistics were used to describe maternal baseline characteristics, rate of GWG, incidence of GDM, and glycemic values. Differences in these variables were compared between participants with and without available dietary data by the independent sample t-test or chi-squared test. Among those with dietary data, differences in maternal characteristics, incidence of GDM, and glycemic concentrations across AHEI-2010 quartiles were determined by one-way ANOVA with *post-hoc* Tukey’s test for continuous variables, and by chi-squared test for categorical variables. In the case of glycemic variables, AHEI-2010 quartiles were computed separately for each subset of participants with available glucose screening and/or tolerance testing data. The association between the continuous AHEI-2010 score and odds of developing GDM and having a raised glucose concentration (≥140 mg/dl) on the 50g glucose screening test were determined by logistic regression. A sensitivity analysis of the association between AHEI-2010 score and the odds of GDM was also performed separately for each group of subjects screened by the 1-step or 2-step methods. Associations between AHEI-2010 total score and component scores with glucose concentrations from the 50g glucose screening test, and with fasting and post glucose load concentrations on each GTT were analyzed by separate linear regression models. Missing data for covariates were handled by pairwise deletion in regression models. Results were considered statistically significant at p<0.05.

## Results

**Figure 1:**
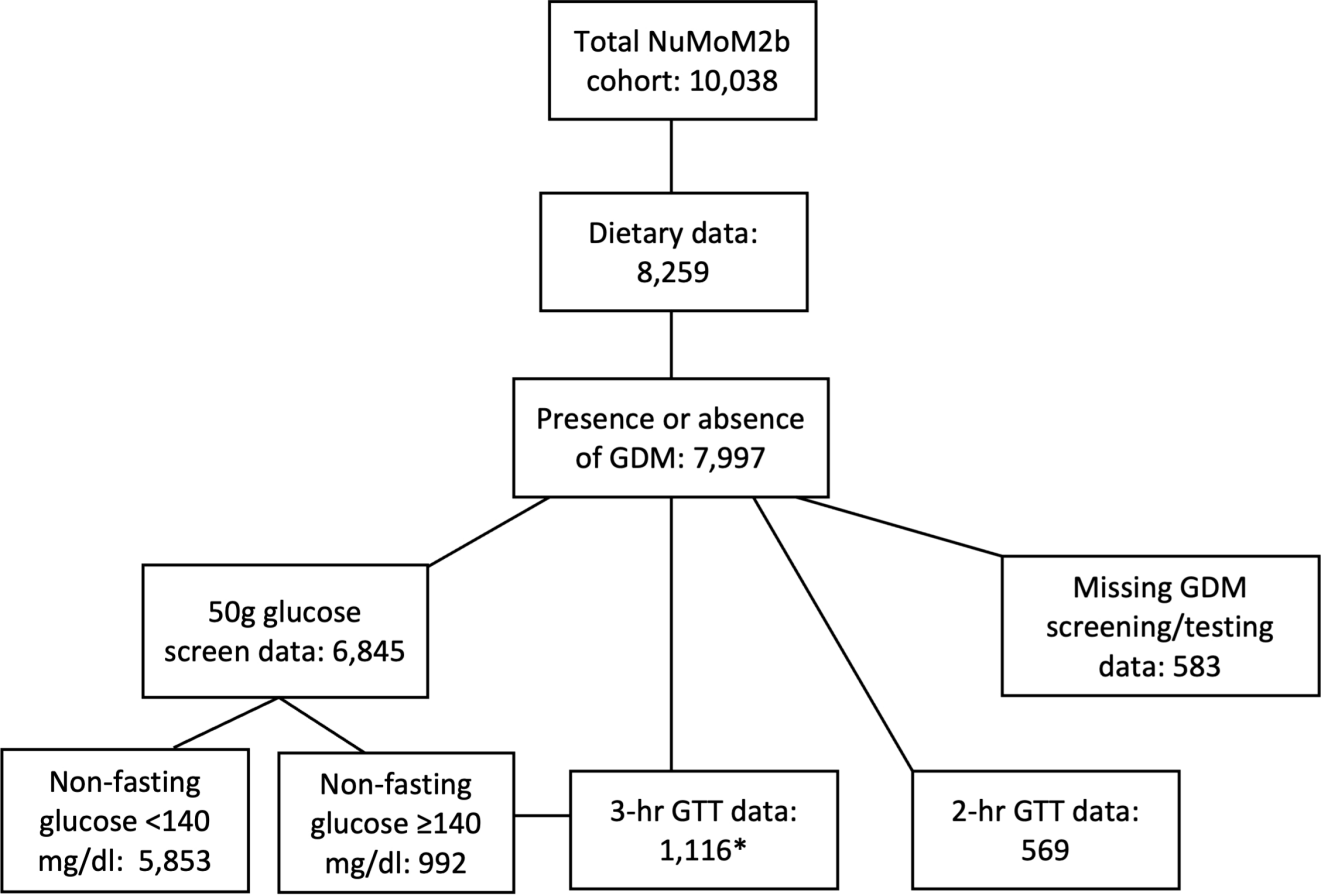
Flowchart of participants for the present analysis. Number of study participants with available data for presence or absence of GDM, diet quality, GDM screening, and GDM diagnostic testing. *3-hr GTT data available for 992 participants with a raised glucose concentration on the 50g screening test, plus 124 additional participants with other GDM risk factors who did not undergo a screening test. GDM, gestational diabetes mellitus; GTT, glucose tolerance test; NuMoM2b, Nulliparous Pregnancy Outcomes Study: Monitoring Mothers-To-Be.

There were 10,038 participants in the parent NuMoM2b study, 8259 of whom had dietary information from which AHEI-2010 scores were computed. Among these, 7997 had a documented outcome for GDM (presence or absence) and were included in the present study. **Figure 1** describes the number of participants with available data according to each stage of GDM screening and/or diagnostic testing. Among those undergoing the 2-step testing method, 992 were at risk for GDM and underwent a 3-hour GTT. An additional 124 individuals underwent a 3-hour GTT who did not have the 50g screening test performed, presumably due to other risk factors (e.g., family history of diabetes, raised hemoglobin A1c value). A total of 325/7997 participants (4.1%) were documented as having a diagnosis of GDM. Of those, 42 did not have any recorded GTT data.

NuMoM2b participants with missing dietary data were, on average, of younger age, higher BMI, with a lower education and income level, and more likely to smoke periconceptionally and to be of Black or Hispanic versus White ethnicity (**Supplemental Table 1**). However, there were no significant differences in the incidence of GDM or having a raised blood glucose concentration on the screening test between those with missing and available dietary data.

Descriptive data for the study population stratified by AHEI-2010 quartile are presented in **Table 1**. All maternal demographic characteristics were significantly associated with periconceptional diet quality. Specifically, women of younger age, higher early-pregnancy BMI, lower educational attainment, periconceptional smoking habits, and below the federal poverty threshold were more likely to have an AHEI-2010 score in the lower quartiles (**Table 1**). Women from minority groups (Black and Hispanic participants) were also more likely to have a poorer quality diet compared to women of Non-Hispanic White or Asian race/ethnicity. Although the rate of GWG per week was significantly higher in those with AHEI-2010 scores in the highest versus lowest quartiles on ANOVA testing, this difference was no longer significant after adjusting for early-pregnancy BMI.

**Table 1:** Descriptive statistics of study population stratified by periconceptional diet quality.

Maternal characteristic	N with available data	Study population	AHEI Q1	AHEI Q2	AHEI Q3	AHEI Q4	P-value
AHEI-2010 score (range given in parentheses)	7997	55.1 ± 12.5 (22.4 - 96.9)	39.7 ± 4.5^a^ (22.4 - 45.7)	50.2 ± 2.5^b^ (45.8 - 54.5)	58.9 ± 2.6^c^ (54.6 - 63.6)	71.7 ± 6.2^d^ (63.7 - 96.9)	<0.001
Energy intake (Kcal)	7997	1715.2 ± 954.2	1947.8 ± 939.8	1692.6 ± 1010.5	1660.0 ± 1139.7	1561.5 ± 600.0	<0.001
Gestational age at enrolment (weeks)	7944	12.5 ± 2.7	12.5 ± 2.8	12.5 ± 1.9	12.6 ± 2.7	12.6 ± 3.2	0.615
Maternal age (years)	7995	27.3 ± 5.5	23.7 ± 5.1^a^	26.2 ± 5.3^b^	28.6 ± 5.0^c^	30.6 ± 4.3^d^	<0.001
Early pregnancy BMI (kg/m^2^)	7890	26.2 ± 6.2	27.2 ± 7.2^a^	26.8 ± 6.4^a^	26.0 ± 5.8^b^	24.8 ± 4.9^c^	<0.001
BMI category	7890						
Underweight		179 (2.2)	70 (3.6)	43 (2.2)	30 (1.5)	36 (1.8)	<0.001
Normal weight		4066 (50.5)	880 (44.6)	903 (46.1)	1058 (53.5)	1225 (61.8)	
Overweight		1957 (24.5)	460 (23.3)	533 (27.2)	495 (25.0)	469 (23.7)	
Obese class I		922 (11.5)	268 (13.6)	267 (13.6)	231 (11.7)	156 (7.9)	
Obese class II or higher		766 (9.6)	293 (14.9)	214 (10.9)	164 (8.3)	95 (4.8)	
Race/ethnicity	7995						
Non-Hispanic White		5037 (63.2)	965 (48.4)	1168 (58.5)	1384 (69.1)	1540 (76.8)	<0.001
Non-Hispanic Black		906 (11.3)	479 (24.0)	249 (12.5)	122 (6.1)	56 (2.8)	
Hispanic		1317 (16.5)	398 (20.0)	440 (22.0)	289 (14.4)	190 (9.5)	
Asian		336 (4.2)	20 (1.0)	49 (2.5)	131 (6.5)	136 (6.8)	
Other		379 (4.7)	131 (6.6)	90 (4.5)	76 (3.8)	82 (4.1)	
Highest education received	7994						
Some or completed high school		1412 (17.7)	782 (39.2)	407 (20.4)	182 (9.1)	41 (2.0)	<0.001
Some or completed college		4626 (57.8)	1115 (54.9)	1327 (64.5)	1287 (60.6)	1030 (65.0)	
Postgraduate education		1956 (24.5)	116 (5.8)	302 (15.1)	606 (30.3)	932 (46.5)	
Below federal poverty threshold	6647	955 (11.9)	471 (34.2)	267 (16.9)	154 (8.6)	63 (3.3)	<0.001
Smoked tobacco prior to pregnancy	7991	1336 (16.7)	601 (30.2)	366 (18.4)	236 (11.8)	133 (6.6)	<0.001
Rate of GWG (kg/week)	6292	1.1 ± 0.5	1.0 ± 0.6^a^	1.1 ± 0.5	1.1 ± 0.6	1.1 ± 0.4^b^	0.024
Incidence of GDM	7997	326 (4.1)	89 (4.5)	82 (4.1)	85 (4.2)	70 (3.5)	0.447
Elevated 1-hr glucose concentration on 50g screening test	6845	992 (12.7)	261 (15.3)	222 (13.0)	258 (15.1)	251 (14.7)	0.214

Data are presented as mean ± SD for continuous variables or N (%) for categorical variables, where percentage values represent the incidence of a given characteristic within each AHEI quartile. P < 0.05 indicates significant differences across AHEI quartiles, computed by one-way ANOVA for continuous variables or chi-squared test for categorical variables. Different superscript letters indicate significant difference between specific AHEI quartiles for continuous variables, assessed by post-hoc Tukey’s test. AHEI, Alternative Healthy Eating Index; GDM, gestational diabetes mellitus; GWG, gestational weight gain.

The overall rate of GDM was 4%, diagnosed at a mean gestational age of 28 ± 3 weeks among those undergoing the 3-hour 100g GTT, and 26 ± 3 weeks among those undergoing the 2-hour 75g GTT. The incidence of GDM and having an elevated glucose concentration on the 50g screening test did not differ across AHEI-2010 quartiles in the unadjusted analysis (**Table 1**). However, on logistic regression analysis adjusting for covariates, each 1-point increase in the AHEI-2010 score was associated with 0.01 reduced odds of having a GDM diagnosis (B=-0.014, p=0.023, aOR=0.986, 95% CI=0.974 to 0.998). This suggests that a 10-point increase in the AHEI-2010 score periconceptionally would correspond to a 10% reduction in the odds of developing GDM. For example, a 10-point score increase could be achieved by increasing vegetable consumption from two to five portions per day plus increasing fruit consumption from two to four portions. Consuming zero sugar sweetened beverages per day (versus any) or consuming at least one portion of nuts or legumes would also confer a 10-point increase in AHEI-2010 score. In the sensitivity analysis, the significant association between diet quality and odds of GDM among those tested by the 3-hour GTT remained (B=-0.018, p=0.029, aOR=0.983, 95% CI=0.967 to 0.998), while the association among those tested by the 2-hour GTT was not significant (B=-0.015, p=0.473, aOR=0.985, CI=0.946 to 1.026). The association between AHEI-2010 and having a blood glucose concentration ≥140 mg/dl on the screening test did not reach significance (B=-0.006, p=0.092, aOR=0.996, 95% CI=0.986 to 1.001).

**Figure 2:**
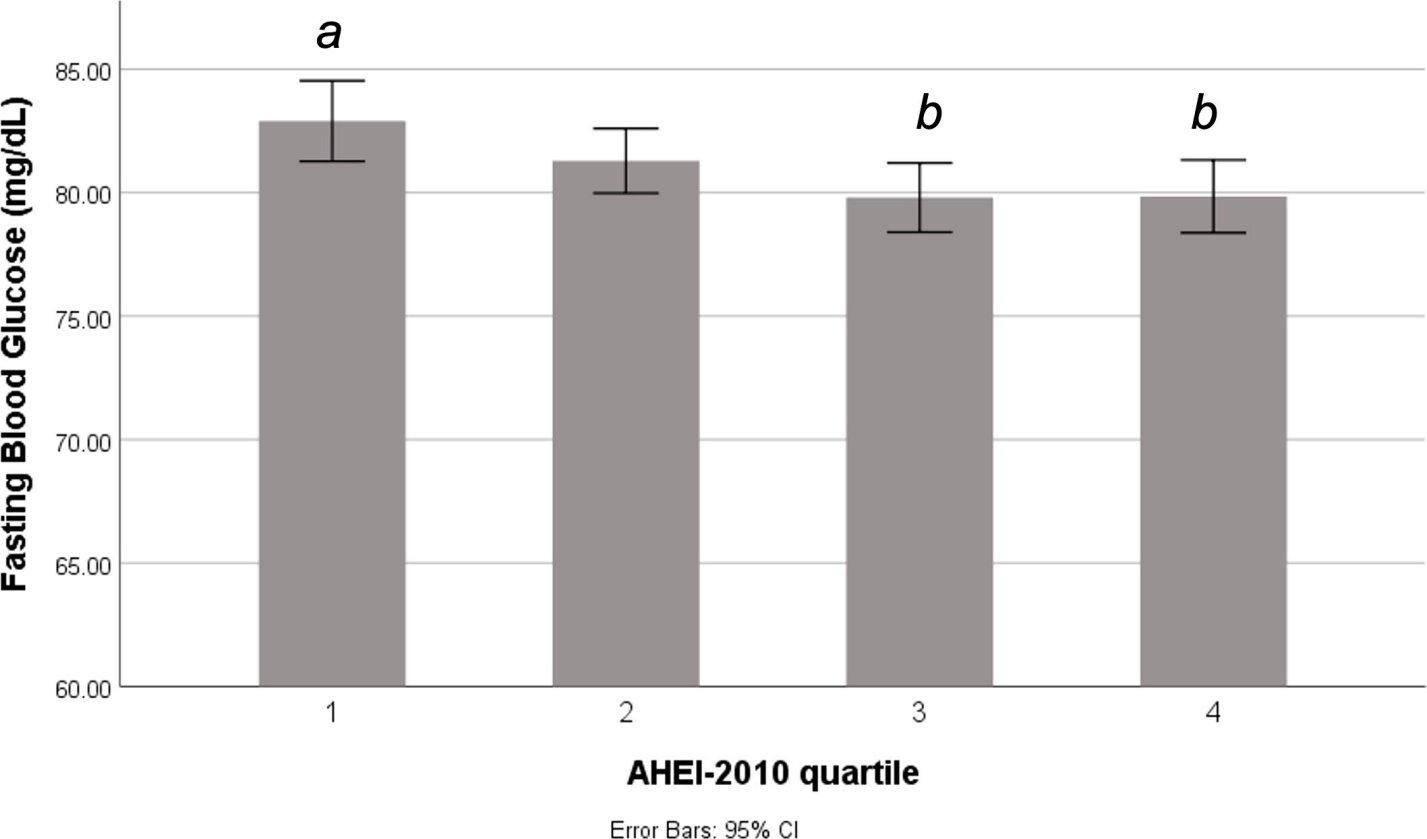
Fasting blood glucose according to periconceptional diet quality among pregnant women at risk for GDM. Difference in fasting blood glucose concentrations across quartiles of the AHEI-2010 score among individuals who underwent a 3-hour glucose tolerance test. Different letters indicate significant difference between quartiles at p<0.05. AHEI, Alternative Healthy Eating Index.

Of those considered at risk for GDM who underwent the 3-hour 100g GTT, fasting glucose concentrations were significantly different across AHEI-2010 quartiles (**Table 2**), such that those in Q3 and Q4 (highest diet quality scores) had slightly lower fasting glucose concentrations than those in Q1 (**Figure 2**). There was no significant difference in mean post glucose load blood glucose concentrations according to AHEI-2010 quartiles on the 3-hour 100g GTT. Among those of all GDM risk levels undergoing the 2-hour 75g GTT, fasting glucose was not significantly different across AHEI-2010 quartiles, although small differences were observed in the 2-hour post glucose load concentrations (**Table 2**).

**Table 2:** Blood glucose concentrations from GDM screening and diagnostic tests stratified by AHEI-2010 quartile*.

Test type	Glucose (mg/dl)	AHEI Q1	AHEI Q2	AHEI Q3	AHEI Q4	P-value
**50g glucose challenge test (N = 6845)**						
1-hr blood glucose	110.8 ± 29.1	110.9 ± 27.5	109.3 ± 27.5	111.9 ± 28.1	111.0 ± 30.6	0.075
**3-hr 100g GTT (N = 1116)**						
Fasting blood glucose	81.0 ± 12.4	82.9 ± 13.7^a^	81.3 ± 11.2	79.8 ± 11.9^b^	79.8 ± 12.5^b^	0.009
1-hr blood glucose	155.5 ± 31.8	158.1 ± 13.7	153.8 ± 31.0	157.6 ± 31.3	152.6 ± 30.6	0.105
2-hr blood glucose	136.5 ± 31.8	137.5 ± 33.3	136.9 ± 31.2	139.1 ± 31.7	132.6 ± 30.8	0.100
3-hr blood glucose	106.3 ± 30.8	107.8 ± 31.3	107.4 ± 30.1	106.8 ± 29.4	103.3 ± 32.1	0.287
**2-hr 75g GTT (N = 569)**						
Fasting blood glucose	75.4 ± 7.7	76.4 ± 7.5	75.4 ± 7.4	75.5 ± 8.5	74.4 ± 7.1	0.178
1-hr blood glucose	118.8 ± 29.6	121.5 ± 28.9	121.6 ± 30.0	118.9 ± 28.8	113.4 ± 30.0	0.065
2-hr blood glucose	102.3 ± 23.9	104.1 ± 24.0	104.5 ± 24.6^a^	97.8 ± 22.0^b^	98.7 ± 24.2	0.027

*AHEI-2010 quartiles computed separately for each subset of participants according to GDM screening or diagnostic testing method. Different superscript letters indicate significant difference between specific AHEI quartiles, assessed by post-hoc Tukey’s test. AHEI, Alternative Healthy Eating Index; GTT, glucose tolerance test.

In the unadjusted linear regression analysis, the total AHEI-2010 score was significantly inversely associated with fasting glucose among those at risk for GDM (B=-0.094, p=0.002), as well as among those of all GDM risk levels undergoing the 2-hour GTT (B=-0.063, p=0.022). Diet quality was not associated with glucose concentrations following the 50g glucose screening test (B=-0.068, p=0.634), but was inversely associated with the 3-hour glucose concentration on the 3-hour GTT (B=-0.156, p=0.035), and with the 1-hour and 2-hour glucose concentrations from the 2-hour GTT (B=-0.224, p=0.021 and B=-0.181, p=0.034, respectively). After adjusting for covariates, all post-glucose load glucose concentrations were significantly inversely associated with the total AHEI-2010 score among those at GDM risk, and the association with fasting glucose trended towards significance (**Table 3**). Among those at all GDM risk levels, diet quality was significantly inversely associated with 1-hour glucose on the 50g screening test, and with 1-hour and 2-hour glucose on the 2-hour GTT, but not with fasting glucose (**Table 3**).

**Table 3:** Associations of periconceptional diet quality (total AHEI-2010 scores) with gestational glucose concentrations on GDM screening and diagnostic testing.

Test type	Beta	Std. Error	P-value	95% CI		Adj R^2^
**50g glucose challenge test**						
1-hr blood glucose	-0.108	0.034	0.001	-0.174	-0.041	0.054
**3-hr 100g GTT**						
Fasting blood glucose	-0.062	0.034	0.069	-0.130	0.005	0.112
1-hr blood glucose	-0.247	0.090	0.006	-0.424	-0.071	0.027
2-hr blood glucose	-0.243	0.090	0.007	-0.420	-0.065	0.025
3-hr blood glucose	-0.228	0.087	0.009	-0.400	-0.056	0.015
**2-hr 75g GTT**						
Fasting blood glucose	-0.072	0.050	0.147	-0.170	0.026	0.092
1-hr blood glucose	-0.374	0.153	0.015	-0.675	-0.073	0.082
2-hr blood glucose	-0.354	0.137	0.010	-0.625	-0.084	0.097

Adjusted for covariates: maternal age, race/ethnicity, smoking status, early pregnancy body mass index, rate of gestational weight gain, energy intake, nausea and vomiting of pregnancy (PUQE score), study site. AHEI, Alternative Healthy Eating Index; GTT, glucose tolerance test.

We explored the associations between AHEI-2010 component scores and glucose results on GDM screening and diagnostic tests, adjusting for confounding factors (**Supplemental Table 2**). Higher intake of long-chain omega-3 fats, primarily found in oily fish, was significantly inversely associated with 1-hour post-glucose load glucose concentration on the non-fasting screening test (B=-0.274, p=0.028), and with fasting (B=-0.265, p=0.036) and 3-hour post glucose load concentration (B=-0.788, p=0.014) on the 3-hour 100g GTT. A 1-point increase in the AHEI-2010 component score for long-chain omega-3 fats can be achieved by a 25 mg increase of fish oil intake per day, which equates to approximately 0.05 oz of wild salmon. Intake of sugary beverages was also independently associated with fasting glucose concentrations on the 3-hour 100g GTT (B=-0.239, p=0.028), such that greater adherence to guidelines to consume zero sugary beverages per day was associated with lower fasting glucose. In general, greater compliance to dietary recommendations for intakes of whole fruit and nuts and legumes was associated with lower post-glucose load glucose concentrations on the 3-hour GTT, while greater compliance to recommended intakes of whole fruit and vegetables was associated with lower post-glucose load concentrations on the 2-hour GTT.

## Discussion

This study presents a detailed analysis of the association between periconceptional diet quality and maternal glycemia in the late second or early third trimester of pregnancy among a large, nationally representative cohort of nulliparous individuals in the U.S. The results indicate that poorer diet quality is associated with increased odds of receiving a GDM diagnosis among those deemed to be at elevated risk of GDM (following the 2-step testing method), as well as having slightly higher glucose concentrations on GDM screening and diagnostic tests among those at all levels of GDM risk. Specifically, each 10-point increase in the AHEI-score, which can be practically achieved through simple dietary modifications such as cutting out sugar sweetened beverages or adding a daily portion of nuts or legumes, could confer a 10% reduced odds of developing GDM. Importantly, these results are independent of established GDM risk factors such as maternal BMI and rate of GWG. Higher intake of sugary beverages and lower intakes of oily fish were most prominently associated with higher glycemic results on the GTT among those at risk for GDM. Women of lower socioeconomic status and with higher BMI were identified as having the lowest periconceptional diet quality, which may contribute to increased risk for impaired gestational glycemia in these maternal populations. However, the NuMoM2b cohort was missing dietary data from some of the most demographically vulnerable women enrolled in the study, which limits the generalizability of these findings.

Few published observational studies exist that report prospective associations of periconceptional diet quality with GDM risk. Consistent with our findings, Tobias et al. reported a significantly reduced risk of GDM with higher AHEI-2010 scores among 15,254 participants of the Nurse’s Health Study II in the U.S (25).. Conversely, in a smaller U.S cohort (N=1733) from the Project Viva study, dietary patterns, glycemic load, and intakes of specific nutrients or food groups in early pregnancy were not associated with GDM or impaired glucose tolerance, with the exception of an unexpected increased risk of GDM with higher omega-3 fatty acid intakes (26). The authors of the Project Viva study concluded that maternal pre-pregnancy BMI was a more significant driver of glycemia than diet. However, in that study a comprehensive diet quality score such as the AHEI-2010 was not utilized and therefore, the smaller sample size may have been insufficient to detect effects of specific dietary components on GDM incidence. Although Yee et al. previously reported no association of periconceptional diet quality measured by the HEI-2010 on GDM risk in the nuMoM2b cohort (17), the AHEI-2010 is a stronger predictor of diabetes risk in non-pregnant adults which may explain this discrepancy (18, 27). The previous study also did not investigate the association of diet quality with glucose concentrations on GDM screening and diagnostic tests, yet elevated glycemic concentrations that do not meet diagnostic criteria for GDM have been associated with an increased risk of adverse pregnancy outcomes. Although we identified some statistically significant differences in glucose concentrations across AHEI-2010 quartiles, these differences were small and may not translate to clinically meaningful perinatal outcomes beyond that of GDM incidence. Regardless, the current study contributes to the existing literature by considering the association of periconceptional diet quality with glycemic concentrations on a continuous spectrum irrespective of GDM diagnosis, and exclusively among a nulliparous cohort who had no prior GDM exposure.

There is increasing recognition that prenatal dietary and lifestyle interventions that are typically initiated around 12-16 weeks’ gestation, are likely too late to exert significant metabolic change or reduced risk of GDM (15, 16). This is particularly relevant among those with pre-pregnancy overweight and obesity who may experience low-grade insulin resistance prior to conception (28). Yet, there is a paucity of published research reporting preconception dietary interventions with follow up across pregnancy, although these are plausible pathways by which a dietary intervention may help to improve gestational glycemia. For example, a few studies of preconception lifestyle interventions involving caloric restriction, physical activity, and behavior modification to achieve weight loss goals among women with obesity and fertility issues have reported beneficial effects on cardiometabolic health, including reductions in BMI, insulin resistance, and the metabolic syndrome (29–31).

Nearly fifty percent of pregnancies in the U.S. are unplanned, leaving many women without time to consider the importance of preconception diet quality (32). Thus, targeted public health initiatives and distribution of resources to support improved diet among non-pregnant women of reproductive age are warranted to help optimize maternal glycemia in future potential pregnancies and reduce the burden of GDM (15, 16, 33). Although women may be more receptive to health behavior change during pregnancy (34), it takes time to establish and maintain the optimal dietary changes that are required to beneficially impact glucose-insulin metabolism. Thus, starting this process around the second trimester is likely too late to substantially benefit gestational metabolic health. In contrast, implementing healthy lifestyle behaviors that help to optimize diet quality and weight status prior to conception may set the stage for easier maintenance of higher quality diet throughout pregnancy.

Results of our study among nulliparous individuals also highlight the population demographics that are most at risk for poor dietary quality. Allocating resources that support healthy dietary behaviors in younger women of reproductive age, who may not even be considering pregnancy in the immediate term, could benefit the health of future maternal populations (15, 35). Clinical interactions with younger, nulliparous women for contraceptive counselling or at well-women visits represents a window of opportunity to initiate conversations around healthy lifestyles for the long-term health benefits for them and for potential future children. Such interactions may require a multidisciplinary approach that includes primary care providers, pediatricians, gynecologists, and registered dietitians. Targeted nutrition education and messaging in schools and colleges could also reach a wide audience for promotion of preventative women’s health. Simple, consistent nutrition messaging is required, such as encouraging avoidance of sugar sweetened beverages, consuming 1-2 portions of oily fish per week, and consuming at least 2.5 cups of vegetables, to support improvements in diet quality for all individuals who could become or currently are pregnant. Whether widespread achievement of these healthy dietary practices could translate to reduced population incidence of GDM remains to be determined.

Nutrition services that support socioeconomically disadvantaged groups are particularly warranted. In the U.S., programs such as Women Infants and Children offer food access and nutrition education only to women who are already pregnant or have young children. Therefore, underserved, nulliparous women who would be eligible for such programs but are not yet pregnant may fall through the gaps. This is a missed opportunity to support the health and wellbeing of our future prenatal populations and their offspring.

Future research directions should include well-designed clinical trials of pre-pregnancy diet and lifestyle interventions to test the effects on gestational glycemia, GDM risk, and other pregnancy complications. The optimal content and mode of delivery for such interventions remains to be determined, but utilizing behavior change theories in the study design, addressing the social determinants of health, and use of multicomponent interventions is recommended.

While our study results may not be generalizable to multiparas, this study is strengthened by its large sample size and diversity in maternal characteristics. Data on the outcome measures, gestational glycemia and GDM diagnosis, were abstracted from medical records by certified chart abstractors at each site. However, there was no harmonization of glucose assay methods across sites for standard GTTs which may be a limitation, although we included study site as a covariate in analyses. Diet was assessed early in gestation using a validated FFQ that captured the periconceptional period, which is frequently not assessed in prenatal studies. As with all retrospective nutrition assessments, the FFQ method is subject to recall bias and misreporting. The absence of dietary assessment later in gestation may be considered a limitation of the study, as it is possible that diet quality scores remain consistent from periconception throughout pregnancy, and diet quality measured in the second trimester may be more strongly associated with odds of GDM and glucose concentrations. However, prenatal dietary interventions initiated in the early second trimester demonstrate inconsistent and low quality evidence for a reduced risk of GDM (36). Given that we found the odds of GDM is already associated with diet quality at the time of periconception, it stands to reason that earlier intervention may help establish healthy glucose tolerance in pregnancy to potentially lessen the likelihood of later GDM development. Another strength is the use of the AHEI-2010, which is considered a more comprehensive tool than the standard HEI to characterize diet quality and its relation to chronic disease risk (18, 19). Lastly, we considered key covariates in our analyses including race/ethnicity, early-pregnancy BMI and rate of GWG, which are recognized as among the most important risk factors for GDM.

In conclusion, a poorer periconceptional diet is independently associated with increased odds of GDM and slightly higher fasting and post glucose load blood glucose concentrations at the time of GDM screening and diagnostic testing in nulliparous individuals. Periconception intervention studies targeting diet quality with prospective follow-up across pregnancy are warranted.

## Data availability statement

Publicly available datasets were analyzed in this study. This data can be found here: NICHD Data and Specimen Hub: https://dash.nichd.nih.gov/explore/study?q=numom2b&filters=[]&page=1&sortBy=relevance&asc=true&size=50.

## Ethics statement

The studies involving human participants were reviewed and approved by each study site’s local governing Institutional Review Board(s) approved the nuMoM2b protocol and procedures: Case Western University; Columbia University; Indiana University; University of Pittsburgh; Northwestern University; University of California at Irvine; University of Pennsylvania; and University of Utah. The patients/participants provided their written informed consent to participate in this study.

## Author contributions

WG, DH, BM, HS, GS, RS, and JC designed research; WG, DH, BM, HS, GS, RS, conducted research; KL analyzed the data; KL and GM wrote the paper; KL and JC had primary responsibility for final content. All authors read and approved the final manuscript.

## Funding

KL was supported by the *Eunice Kennedy Shriver* National Institute of Child Health and Human Development grant R00 HD096109 at the time the study was performed. Support for the NuMoM2b study was provided by grant funding from the *Eunice Kennedy Shriver* National Institute of Child Health and Human Development: RTI International grant U10 HD063036; Case Western Reserve University grant U10 HD063072; Columbia University grant U10 HD063047; Indiana University grant U10 HD063037; University of Pittsburgh grant U10HD063041; Northwestern University grant U10 HD063020; University of California, Irvine grant U10 HD063046; University of Pennsylvania grant U10 HD063048; and University of Utah grant U10 HD063053. In addition, support was provided by respective Clinical and Translational Science Institutes to Indiana University (grant UL1TR001108) and University of California, Irvine (grant UL1TR000153).

## Conflict of interest

The authors declare that the research was conducted in the absence of any commercial or financial relationships that could be construed as a potential conflict of interest.

## Publisher’s note

All claims expressed in this article are solely those of the authors and do not necessarily represent those of their affiliated organizations, or those of the publisher, the editors and the reviewers. Any product that may be evaluated in this article, or claim that may be made by its manufacturer, is not guaranteed or endorsed by the publisher.
