# Supplementary material for: Periconceptional diet quality is associated with gestational diabetes risk and glucose concentrations among nulliparous gravidas
Source: Front Endocrinol (Lausanne). 2022 Sep 5;13:940870. doi: 10.3389/fendo.2022.940870 (PMC9483841; doi:10.3389/fendo.2022.940870)
Supplement: Supplementary file 1 [file Table_1.docx]

| **Supplemental Table 1: Comparison of maternal characteristics and incidence of GDM between those with missing and available dietary data** | | | |
| --- | --- | --- | --- |
| **Maternal characteristic** | **Missing dietary data (N=1779)** | **Available dietary data (N=7997)** | **P-value** |
| Gestational age at enrolment (weeks) | 12.4 ± 3.2 | 12.5 ± 2.7 | 0.040 |
| Maternal age (years) | 25.3 ± 5.8 | 27.3 ± 5.5 | <0.001 |
| Early pregnancy BMI (kg/m^2^) | 27.1 ± 6.8 | 26.2 ± 6.2 | <0.001 |
| BMI category |  |  |  |
| Underweight | 34 (2.3) | 179 (2.2) | <0.001 |
| Normal weight | 689 (46.4) | 4066 (50.5) |  |
| Overweight | 369 (24.8) | 1957 (24.5) |  |
| Obese class I | 206 (13.9) | 922 (11.5) |  |
| Obese class II or higher | 187 (12.6) | 766 (9.6) |  |
| Race/ethnicity |  |  |  |
| Non-Hispanic White | 703 (44.8) | 5037 (63.2) | <0.001 |
| Non-Hispanic Black | 426 (27.2) | 906 (11.3) |  |
| Hispanic | 284 (18.1) | 1317 (16.5) |  |
| Asian | 48 (3.1) | 336 (4.2) |  |
| Other | 107 (6.8) | 379 (4.7) |  |
| Highest education received |  |  |  |
| Some or completed high school | 466 (29.8) | 1412 (17.7) | <0.001 |
| Some or completed college | 839 (53.7) | 4626 (57.8) |  |
| Postgraduate education | 257 (16.5) | 1956 (24.5) |  |
| Below federal poverty threshold | 264 (23.6) | 955 (11.9) | <0.001 |
| Smoked tobacco prior to pregnancy | 350 (22.3) | 1336 (16.7) | <0.001 |
| Rate of GWG (kg/week) | 1.0 ± 0.6 | 1.1 ± 0.5 | 0.007 |
| Incidence of GDM | 70 (4.5) | 326 (4.1) | 0.484 |
| Elevated 1-hr glucose value on 50g screening test | 173 (13.2) | 992 (12.7) | 0.215 |

Data are presented as mean ± SD for continuous variables or N (%) for categorical variables. P<0.05 indicates significant differences between those with missing versus available dietary data, computed by independent sample t-test for continuous variables or chi-squared test for categorical variables. AHEI, Alternative Healthy Eating Index; GDM, gestational diabetes mellitus; GWG, gestational weight gain.
