# Supplementary material for: Periconceptional diet quality is associated with gestational diabetes risk and glucose concentrations among nulliparous gravidas
Source: Front Endocrinol (Lausanne). 2022 Sep 5;13:940870. doi: 10.3389/fendo.2022.940870 (PMC9483841; doi:10.3389/fendo.2022.940870)
Supplement: Supplementary file 2 [file Table_2.docx]

**Supplemental Table 2: Association of AHEI-2010 components with glucose concentrations on GDM screening and diagnostic tests, adjusted for covariates.**

| **50g Glucose Screening Test** | **Beta** | **Std. Error** | **P-value** | **95% CI** | |
| --- | --- | --- | --- | --- | --- |
| AHEI-2010 Vegetable (not potato) servings score | -0.245 | 0.151 | 0.106 | -0.542 | 0.052 |
| AHEI-2010 Fruit (not juice) servings score | -0.246 | 0.130 | 0.059 | -0.501 | 0.059 |
| AHEI-2010 Whole grain servings score | -0.256 | 0.260 | 0.324 | -0.765 | 0.253 |
| AHEI-2010 Sugary beverages (and juice) servings score | -0.133 | 0.108 | 0.217 | -0.344 | 0.078 |
| AHEI-2010 Nuts and legumes servings score | -0.093 | 0.123 | 0.452 | -0.334 | 0.149 |
| AHEI-2010 Red meats servings score | -0.247 | 0.160 | 0.123 | -0.561 | 0.067 |
| AHEI-2010 Trans-fat percent score | -0.639 | 0.328 | 0.051 | -1.282 | 0.003 |
| AHEI-2010 DHA & EPA (fish fatty acids) intake score | -0.274 | 0.124 | *0.028* | -0.518 | -0.030 |
| AHEI-2010 Polyunsaturated fat (oils) percent score | 0.124 | 0.196 | 0.527 | -0.260 | 0.507 |
| AHEI-2010 Alcoholic drinks score | -0.251 | 0.026 | *0.041* | -0.491 | -0.011 |
| **100g GTT - Fasting Glucose** |  |  |  |  |  |
| AHEI-2010 Vegetable (not potato) servings score | 0.032 | 0.154 | 0.833 | -0.269 | 0.334 |
| AHEI-2010 Fruit (not juice) servings score | -0.095 | 0.132 | 0.472 | -0.355 | 0.164 |
| AHEI-2010 Whole grain servings score | 0.146 | 0.263 | 0.581 | -0.371 | 0.662 |
| AHEI-2010 Sugary beverages (and juice) servings score | -0.239 | 0.109 | *0.028* | -0.453 | -0.025 |
| AHEI-2010 Nuts and legumes servings score | -0.149 | 0.125 | 0.231 | -0.394 | 0.095 |
| AHEI-2010 Red meats servings score | -0.030 | 0.162 | 0.856 | -0.348 | 0.289 |
| AHEI-2010 Trans-fat percent score | 0.254 | 0.332 | 0.445 | -0.398 | 0.906 |
| AHEI-2010 DHA & EPA (fish fatty acids) intake score | -0.265 | 0.126 | *0.036* | -0.512 | -0.018 |
| AHEI-2010 Polyunsaturated fat (oils) percent score | -0.275 | 0.198 | 0.166 | -0.664 | 0.114 |
| AHEI-2010 Alcoholic drinks score | 0.158 | 0.124 | 0.202 | -0.085 | 0.402 |
| **100g GTT - 1-hr Glucose** |  |  |  |  |  |
| AHEI-2010 Vegetable (not potato) servings score | -0.192 | 0.404 | 0.634 | -0.984 | 0.599 |
| AHEI-2010 Fruit (not juice) servings score | -0.991 | 0.346 | *0.004* | -1.670 | -0.312 |
| AHEI-2010 Whole grain servings score | -1.020 | 0.691 | 0.140 | -2.375 | 0.336 |
| AHEI-2010 Sugary beverages (and juice) servings score | -0.276 | 0.287 | 0.337 | -0.839 | 0.287 |
| AHEI-2010 Nuts and legumes servings score | -0.837 | 0.237 | *0.010* | -1.478 | -0.196 |
| AHEI-2010 Red meats servings score | -0.858 | 0.426 | *0.044* | -1.693 | -0.022 |
| AHEI-2010 Trans-fat percent score | -1.412 | 0.872 | 0.106 | -3.123 | 0.300 |
| AHEI-2010 DHA & EPA (fish fatty acids) intake score | -0.232 | 0.331 | 0.484 | -0.882 | 0.418 |
| AHEI-2010 Polyunsaturated fat (oils) percent score | 0.050 | 0.521 | 0.923 | -0.972 | 1.073 |
| AHEI-2010 Alcoholic drinks score | 0.047 | 0.326 | 0.884 | -0.593 | 0.688 |
| **100g GTT - 2-hr Glucose** |  |  |  |  |  |
| AHEI-2010 Vegetable (not potato) servings score | -0.785 | 0.405 | 0.053 | -1.579 | 0.009 |
| AHEI-2010 Fruit (not juice) servings score | -0.742 | 0.348 | *0.033* | -1.425 | -0.059 |
| AHEI-2010 Whole grain servings score | -1.313 | 0.694 | 0.059 | -2.674 | 0.048 |
| AHEI-2010 Sugary beverages (and juice) servings score | -0.285 | 0.288 | 0.323 | -0.850 | 0.280 |
| AHEI-2010 Nuts and legumes servings score | -0.857 | 0.258 | *0.009* | -1.500 | -0.213 |
| AHEI-2010 Red meats servings score | -0.438 | 0.428 | 0.307 | -1.278 | 0.403 |
| AHEI-2010 Trans-fat percent score | -1.235 | 0.876 | 0.159 | -2.954 | 0.485 |
| AHEI-2010 DHA & EPA (fish fatty acids) intake score | -0.316 | 0.333 | 0.342 | -0.969 | 0.336 |
| AHEI-2010 Polyunsaturated fat (oils) percent score | -0.451 | 0.523 | 0.389 | -1.478 | 0.576 |
| AHEI-2010 Alcoholic drinks score | 0.050 | 0.328 | 0.879 | -0.594 | 0.693 |
| **100g GTT - 3-hr Glucose** |  |  |  |  |  |
| AHEI-2010 Vegetable (not potato) servings score | -0.644 | 0.391 | 0.100 | -1.411 | 0.124 |
| AHEI-2010 Fruit (not juice) servings score | -0.737 | 0.336 | *0.029* | -1.397 | -0.077 |
| AHEI-2010 Whole grain servings score | -1.160 | 0.670 | 0.084 | -2.476 | 0.155 |
| AHEI-2010 Sugary beverages (and juice) servings score | -0.408 | 0.278 | 0.143 | -0.954 | 0.138 |
| AHEI-2010 Nuts and legumes servings score | -0.550 | 0.317 | 0.084 | -1.173 | 0.073 |
| AHEI-2010 Red meats servings score | -0.333 | 0.414 | 0.422 | -1.145 | 0.479 |
| AHEI-2010 Trans-fat percent score | 0.058 | 0.847 | 0.946 | -1.605 | 1.720 |
| AHEI-2010 DHA & EPA (fish fatty acids) intake score | -0.788 | 0.321 | *0.014* | -1.417 | -0.159 |
| AHEI-2010 Polyunsaturated fat (oils) percent score | -0.162 | 0.506 | 0.749 | -1.154 | 0.831 |
| AHEI-2010 Alcoholic drinks score | 0.313 | 0.317 | 0.323 | -0.308 | 0.935 |
| **75g GTT – Fasting Glucose** |  |  |  |  |  |
| AHEI-2010 Vegetable (not potato) servings score | -0.055 | 0.223 | 0.807 | -0.494 | 0.385 |
| AHEI-2010 Fruit (not juice) servings score | -0.287 | 0.192 | 0.135 | -0.664 | 0.089 |
| AHEI-2010 Whole grain servings score | -0.512 | 0.382 | 0.180 | -1.263 | 0.238 |
| AHEI-2010 Sugary beverages (and juice) servings score | -0.015 | 0.159 | 0.925 | -0.327 | 0.298 |
| AHEI-2010 Nuts and legumes servings score | 0.057 | 0.981 | 0.754 | -0.300 | 0.413 |
| AHEI-2010 Red meats servings score | -0.161 | 0.236 | 0.495 | -0.625 | 0.303 |
| AHEI-2010 Trans-fat percent score | -0.377 | 0.483 | 0.435 | -1.327 | 0.572 |
| AHEI-2010 DHA & EPA (fish fatty acids) intake score | -0.168 | 0.183 | 0.360 | -0.528 | 0.192 |
| AHEI-2010 Polyunsaturated fat (oils) percent score | 0.085 | 0.288 | 0.770 | -0.483 | 0.652 |
| AHEI-2010 Alcoholic drinks score | -0.105 | 0.181 | 0.562 | -0.460 | 0.250 |
| **75g GTT - 1-hr Glucose** |  |  |  |  |  |
| AHEI-2010 Vegetable (not potato) servings score | -1.382 | 0.685 | *0.045* | -2.731 | -0.034 |
| AHEI-2010 Fruit (not juice) servings score | -1.144 | 0.590 | 0.053 | -2.305 | 0.017 |
| AHEI-2010 Whole grain servings score | -0.226 | 1.182 | 0.849 | -2.551 | 2.100 |
| AHEI-2010 Sugary beverages (and juice) servings score | -0.296 | 0.490 | 0.547 | -1.260 | 0.269 |
| AHEI-2010 Nuts and legumes servings score | -0.551 | 0.559 | 0.325 | -1.651 | 0.549 |
| AHEI-2010 Red meats servings score | -1.057 | 0.727 | 0.147 | -2.486 | 0.372 |
| AHEI-2010 Trans-fat percent score | -0.769 | 1.492 | 0.606 | -3.704 | 2.166 |
| AHEI-2010 DHA & EPA (fish fatty acids) intake score | -0.835 | 0.564 | 0.140 | -1.945 | 0.276 |
| AHEI-2010 Polyunsaturated fat (oils) percent score | -0.645 | 0.890 | 0.469 | -2.396 | 1.106 |
| AHEI-2010 Alcoholic drinks score | -0.191 | 0.558 | 0.732 | -1.289 | 0.906 |
| **75g GTT - 2-hr Glucose** |  |  |  |  |  |
| AHEI-2010 Vegetable (not potato) servings score | -1.429 | 0.615 | *0.021* | -2.640 | -0.219 |
| AHEI-2010 Fruit (not juice) servings score | -1.128 | 0.530 | *0.034* | -2.171 | -0.085 |
| AHEI-2010 Whole grain servings score | -0.288 | 1.063 | 0.787 | -2.379 | 1.803 |
| AHEI-2010 Sugary beverages (and juice) servings score | -0.141 | 0.441 | 0.749 | -1.009 | 0.727 |
| AHEI-2010 Nuts and legumes servings score | -0.322 | 0.503 | 0.522 | -1.312 | 0.667 |
| AHEI-2010 Red meats servings score | -1.115 | 0.653 | 0.089 | -2.399 | 0.169 |
| AHEI-2010 Trans-fat percent score | -2.074 | 1.337 | 0.122 | -4.705 | 0.557 |
| AHEI-2010 DHA & EPA (fish fatty acids) intake score | -0.989 | 0.506 | 0.052 | -1.985 | 0.007 |
| AHEI-2010 Polyunsaturated fat (oils) percent score | -0.388 | 0.801 | 0.629 | -1.963 | 1.188 |
| AHEI-2010 Alcoholic drinks score | 0.276 | 0.502 | 0.583 | -0.711 | 1.262 |

Higher AHEI component scores imply greater adherence to dietary guidelines for the given food group or nutrient, i.e. higher sugary beverage score indicates lower consumption of sugary beverages. Adjusted for covariates: maternal age, race/ethnicity, smoking status, early pregnancy body mass index, rate of gestational weight gain, study site, energy intake, nausea and vomiting in the first trimester. AHEI, Alternative Healthy Eating Index; DHA, docosahexaenoic acid; EPA, eicosapentaenoic acid; GTT, glucose tolerance test.
